# Supplementary material for: Nanopore Direct RNA Sequencing Reveals the Short-Term Salt Stress Response in Maize Roots
Source: Plants (Basel). 2024 Jan 30;13(3):405. doi: 10.3390/plants13030405 (PMC10857558; doi:10.3390/plants13030405)
Supplement: Supplementary file 1 [file plants-13-00405-s001.zip › Supplementary Figure .pdf]

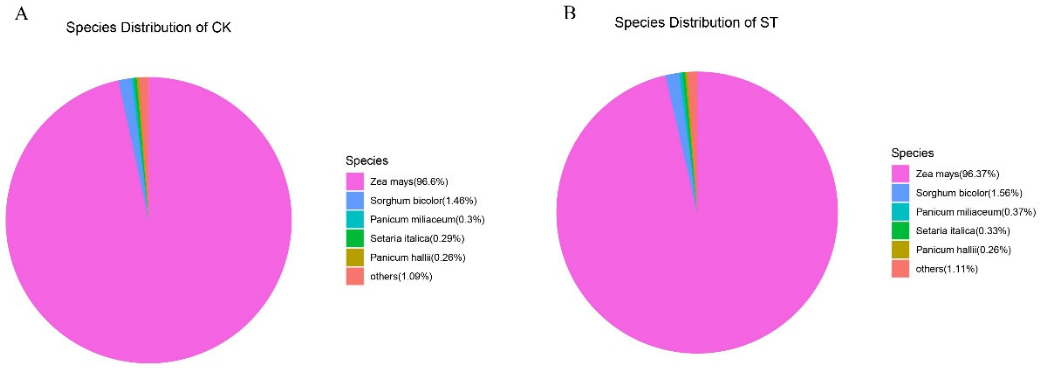

Supplementary Figure S1: Species taxonomy plot of NR annotation

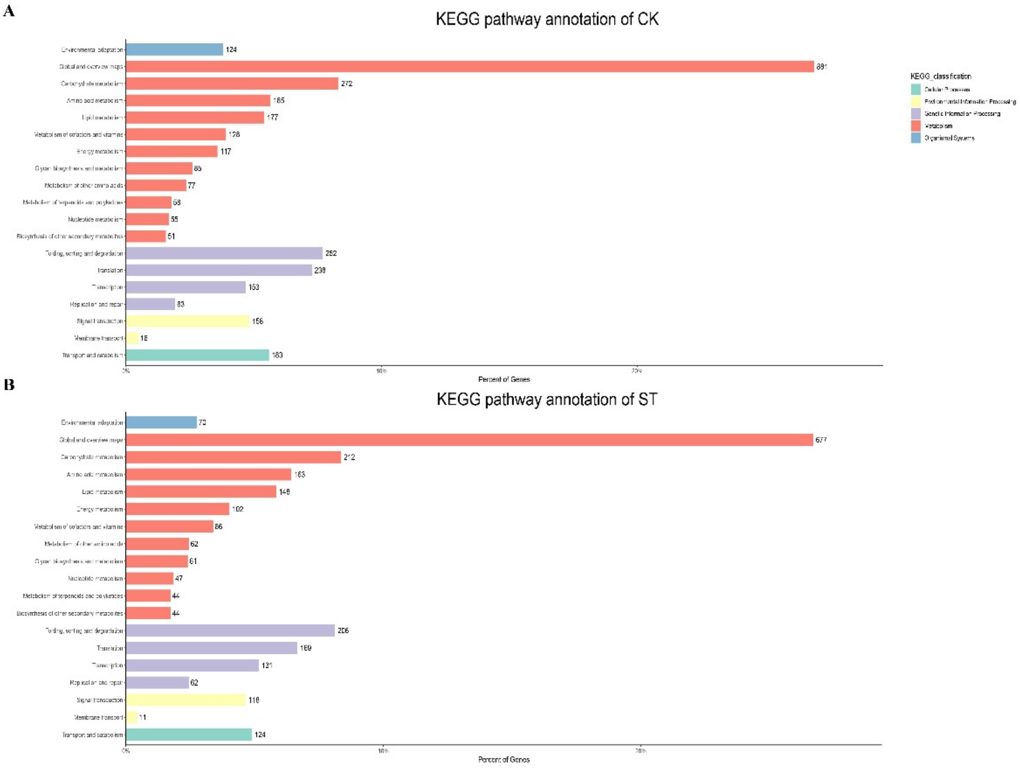

Supplementary Figure S2: KEGG annotation statistics for CK and ST transcripts

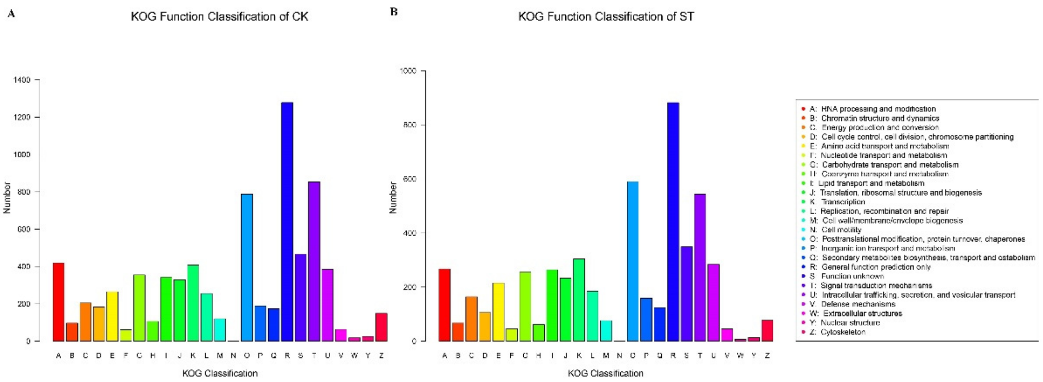

Supplementary Figure S3: KOG annotation statistics for CK and ST transcripts

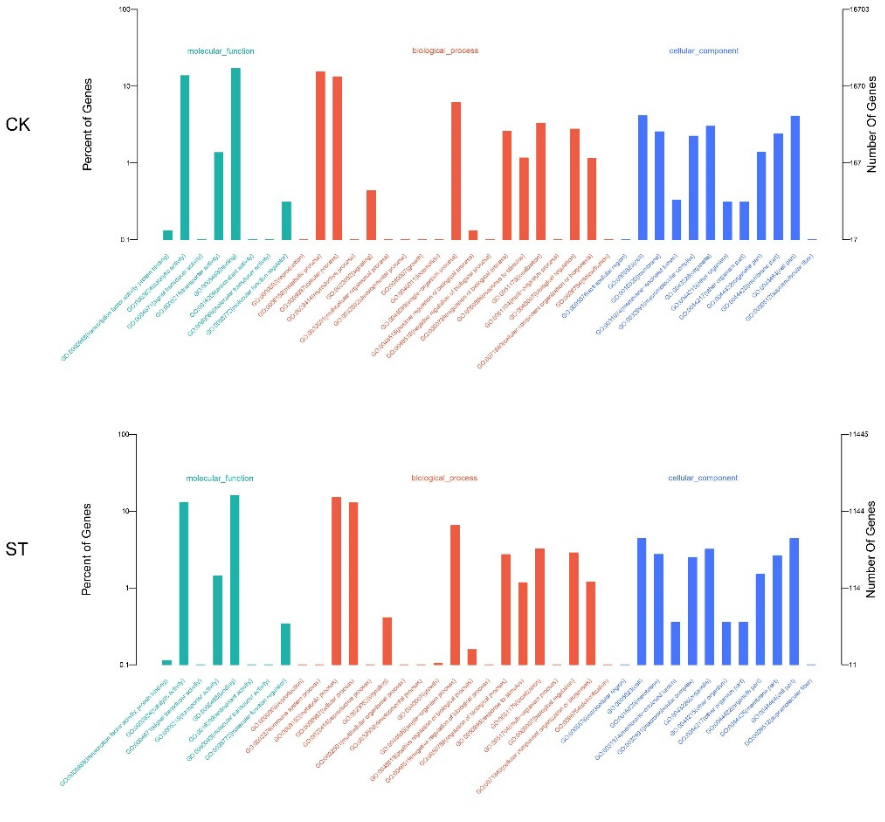

Supplementary Figure S4: GO annotation statistics for CK and ST transcripts

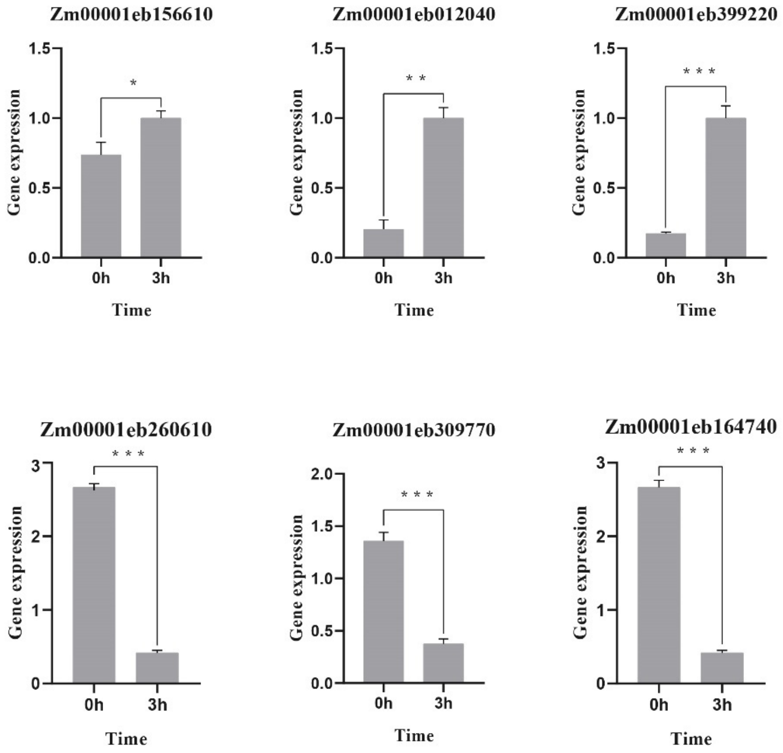

Supplementary Figure S5: The qRT-PCR results figure. “ * ” indicates P<0.05, " ** " indicates P<0.01, and " *** ” indicates P<0.001.
